# Extensive Natural Epigenetic Variation at a *De Novo* Originated Gene

**DOI:** 10.1371/journal.pgen.1003437

**Published:** 2013-04-11

**Authors:** Amanda Bortolini Silveira, Charlotte Trontin, Sandra Cortijo, Joan Barau, Luiz Eduardo Vieira Del Bem, Olivier Loudet, Vincent Colot, Michel Vincentz

**Affiliations:** 1 Centro de Biologia Molecular e Engenharia Genética, Universidade Estadual de Campinas, Cidade Universitária “Zeferino Vaz,” Campinas, São Paulo, Brazil; 2 INRA, UMR 1318, Institut Jean-Pierre Bourgin, Versailles, France; 3 Ecole Normale Supérieure, Institut de Biologie de l'ENS (IBENS), Centre National de la Recherche Scientifique (CNRS) UMR 8197, Institut National de la Santé et de la Recherche Médicale (Inserm) U1024, Paris, France; 4 Departamento de Biologia Vegetal, Universidade Estadual de Campinas, Cidade Universitária “Zeferino Vaz,” Campinas, São Paulo, Brazil; New York University, United States of America

## Abstract

Epigenetic variation, such as heritable changes of DNA methylation, can affect gene expression and thus phenotypes, but examples of natural epimutations are few and little is known about their stability and frequency in nature. Here, we report that the gene *Qua-Quine Starch* (*QQS*) of *Arabidopsis thaliana*, which is involved in starch metabolism and that originated *de novo* recently, is subject to frequent epigenetic variation in nature. Specifically, we show that expression of this gene varies considerably among natural accessions as well as within populations directly sampled from the wild, and we demonstrate that this variation correlates negatively with the DNA methylation level of repeated sequences located within the 5′end of the gene. Furthermore, we provide extensive evidence that DNA methylation and expression variants can be inherited for several generations and are not linked to DNA sequence changes. Taken together, these observations provide a first indication that *de novo* originated genes might be particularly prone to epigenetic variation in their initial stages of formation.

## Author Summary

Epigenetics is defined as the study of heritable changes in gene expression that are not linked to changes in the DNA sequence. In plants, these heritable variations are often associated with differences in DNA methylation. So far, very little is known about the extent and stability of epigenetic variation in nature. In this study, we report a case of extensive epigenetic variation in natural populations of the flowering plant *Arabidopsis thaliana*, which concerns a gene involved in starch metabolism, named *Qua-Quine Starch* (*QQS*). We show that in the wild *QQS* expression varies extensively and concomitantly with DNA methylation of the gene promoter. We also demonstrate that these variations are independent of DNA sequence changes and are stably inherited for several generations. In view of the recent evolutionary origin of *QQS*, we speculate that genes that emerge from scratch could be particularly prone to epigenetic variation. This would in turn endow epigenetic variation with a unique adaptive role in enabling *de novo* originated genes to adjust their expression pattern.

## Introduction

DNA mutations are the main known source of heritable phenotypic variation, but epimutations, such as heritable changes of gene expression associated with gain or loss of DNA methylation, are also a source of phenotypic variability. Indeed, several stable DNA methylation variants affecting a wide range of characters have been described, mainly in plants [1]–[3]. In most instances, epimutations are linked to the presence of structural features near or within genes, such as direct [4]–[6] or inverted repeats [7], [8] or transposable element (TE) insertions [9], which act as units of DNA methylation through the production of small interfering RNAs (siRNAs) [3], [10]. Examples of epimutable loci in *Arabidopsis thaliana* (*A. thaliana*) include the *PAI* [7] and *ATFOLT1* genes [8], which have suffered siRNA-producing duplication events in some accessions and also the well characterized *FWA* locus, which contains a set of SINE-derived siRNA-producing tandem repeats at its 5′end [4], [5]. Repeat-associated epimutable loci are almost invariably found in the methylated form [5]–[9] in nature, which reflects, at least in part, that DNA methylation is particularly well-maintained over repeats [11], [12]. Indeed, epigenetic variation at *PAI*, *ATFOLT1* and *FWA* has only been observed in experimental settings. Similarly, sporadic gain or loss of DNA methylation associated with changes in gene expression has only been documented in *A. thaliana* mutation accumulation lines [13], [14] and examples of natural epigenetic variation in other plant species are few [15]–[17].

Here we report a case of prevalent natural epigenetic variation in *A. thaliana*, which concerns a *de novo* originated gene [18]. We show that expression of this gene, named *Qua-Quine Starch* (*QQS*), is inversely correlated with the DNA methylation level of its promoter and that these variations are stably inherited for several generations, independently of DNA sequence changes. Based on these findings, we speculate that epigenetic variation could be particularly beneficial for newly formed genes, as it would enable them to explore more effectively the expression landscape than through rare DNA sequence changes.

## Results

### *QQS* Is a Novel Gene Embedded within a TE-Rich Region of the *A. thaliana* Genome and Is Negatively Regulated by DNA Methylation

The *A. thaliana Qua-Quine Starch* (*QQS, At3g30720*) was first described as a gene involved in starch metabolism in leaves [19], [20]. Despite being functional and presumably already under purifying selection (dN/dS = 0.5868; p-value<0.045), *QQS* is likely a recent gene that emerged *de novo*. Indeed, *QQS* has no significant similarity to any other sequence present in GenBank [18], [19], suggesting that it originated from scratch since *A.thaliana* diverged from its closest sequenced relative *A. lyrata* around 5–10 million years ago. Furthermore, *QQS* encodes a short protein (59 amino acids) and it is differentially expressed under various abiotic stresses [18], which are also hallmarks of *de novo* originated genes [21]–[23].

**Figure 1:**
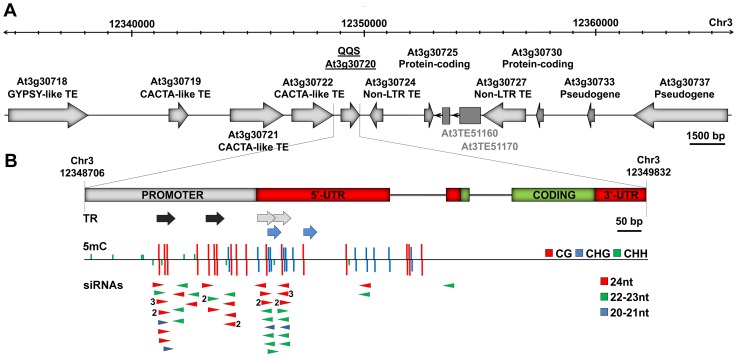
*QQS* is embedded in a repeat-rich region. (A) Genomic structure of the *QQS* locus (30 kb window) in the Col-0 accession. Dark grey boxes represent two additional TE sequences predicted by [51], [52]. (B) Magnified view of the *QQS* gene and upstream sequences, showing tandem repeats (TR), methylation of cytosine residues (5 mC) at the three types of sites (CG, CHG and CHH, H = A, T or C) and locus-specific sense and antisense siRNAs (numbers referring to copy number). DNA methylation and siRNA data are from [25].

As shown in Figure 1, *QQS* is surrounded by multiple transposable element sequences (Figure 1A) and contains several tandem repeats in its promoter region and 5′UTR (Figure 1B). In the Columbia (Col-0) accession, these tandem repeats are densely methylated and produce predominantly 24 nt-long siRNAs (Figure 1B, Figure S1A and S1B). Publically available transcriptome data [24], [25] and results of RT-qPCR analyses (Figure S1C) show that steady state levels of *QQS* mRNAs are higher in several mutants affected in the DNA methylation of repeat sequences, including *met1* (*DNA METHYLTRANSFERASE 1*), *ddc* (*DOMAINS REARRANGED METHYLTRANSFERASE 1 and 2 and CHROMOMETHYLASE 3*), *ddm1* (*DECREASE IN DNA METHYLATION 1*) and *rdr2* (*RNA-DEPENDENT RNA POLYMERASE 2*), which abolishes the production of 24 nt-long siRNAs as well as most CHH methylation. These findings indicate that *QQS* expression is negatively controlled by DNA methylation and point to the siRNA-producing tandem repeats as being potentially involved in this repression.

### Stably Inherited Spontaneous and Induced Epigenetic Variation at *QQS*

**Figure 2:**
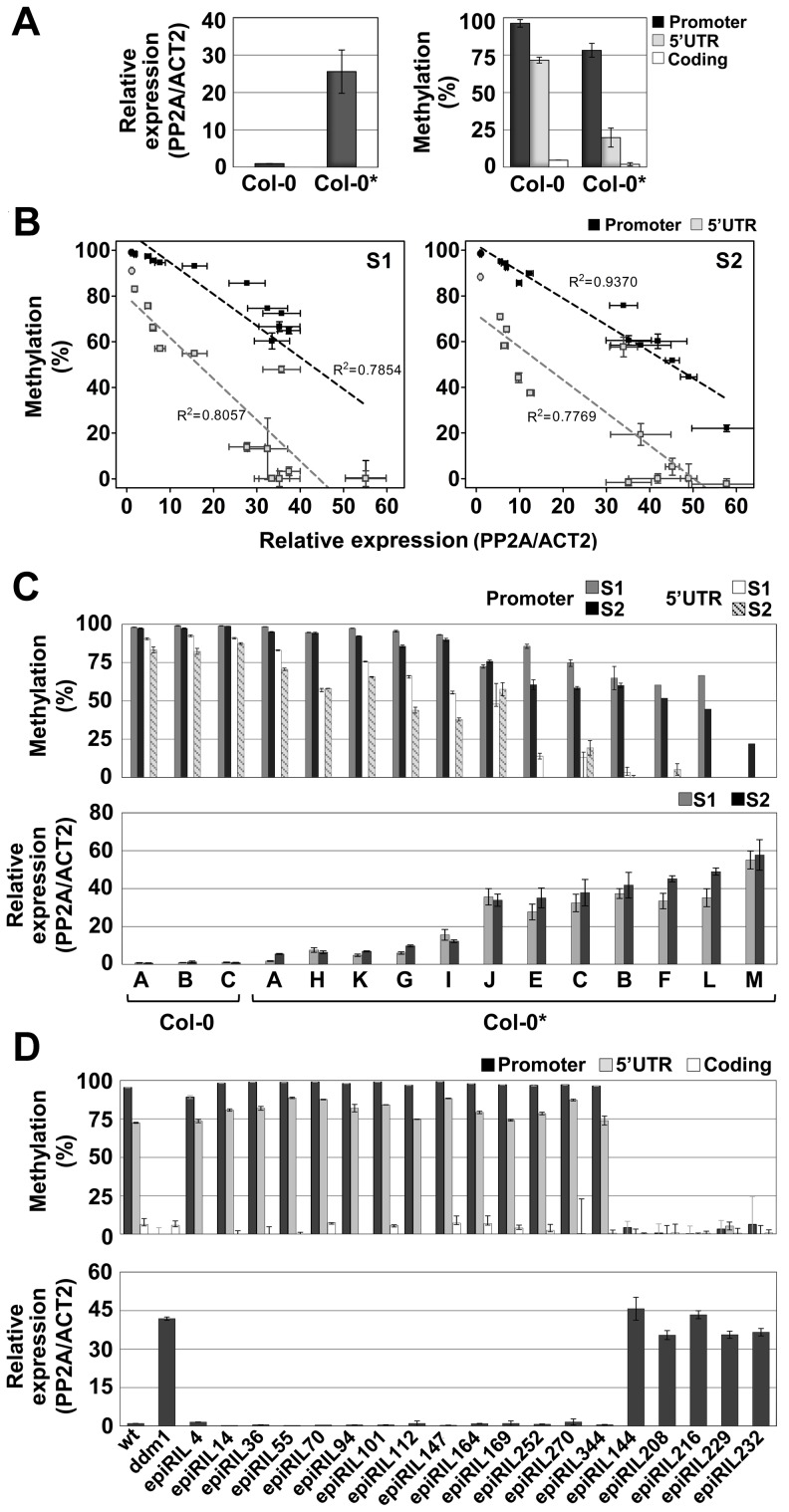
Spontaneous and induced epigenetic variation at *QQS*. (A) DNA methylation and expression profiles of *QQS* in seedlings of the Col-0 and Col-0* stocks. (B) and (C) Negative correlation between *QQS* DNA methylation and expression levels in pooled seedlings of Col-0 and Col-0* (represented by circles and squares in B, respectively) S1 and S2 generation single seed descent lines. (D) DNA methylation and expression levels of *QQS* in seedlings of *ddm1*-derived epiRILs. Error bars represent standard deviation observed in three biological replicates (A–D – expression; A – DNA methylation) or two technical replicates (B–D – DNA methylation).

We first observed epiallelic variation at *QQS* unexpectedly, in a Col-0 laboratory stock (hereafter referred to as Col-0*) with increased expression of the gene and decreased DNA methylation of its promoter and 5′UTR repeat elements (Figure 2A). No sequence change could be detected in the Col-0* stock within a 1.2 kb region covering the *QQS* gene (Figure 1B), which excluded local *cis*-regulatory DNA mutations at the *QQS* locus as being responsible for DNA methylation loss in Col-0*. Additionally, comparative genomic hybridization analysis as well as genome-wide DNA methylation profiling using methylated DNA imunoprecipitation assays revealed no major differences between Col-0 and Col-0* (Figure S2).

We next investigated the *QQS* epigenetic status in pooled seedlings (S1) derived from the selfing of 12 individual Col-0* plants (Figure S3). Results revealed a range of *QQS* epialleles and a strong negative correlation between DNA methylation and expression of the gene (Figure 2B and 2C). To explore further this variation, a single S1 individual was then selfed for each of the 12 lines and seedlings (S2) were analyzed in pool for each line, as above (Figure S3). Remarkably, the differences in *QQS* expression and DNA methylation observed at the S1 generation were also observed at the S2 generation (Figure 2B and 2C). Taken together, these results suggest therefore the existence of a range of epiallelic variants at *QQS*, which are stably inherited for at least one generation.

The inheritance of *QQS* hypomethylated epialleles was also examined in a random sample of 19 *ddm1*-derived epigenetic Recombinant Inbred Lines (epiRILs) obtained by crossing a Col-0 wild-type (wt) line with an hypomethylated Col-0 *ddm1* line [26]. High DNA methylation/low expression and low DNA methylation/high expression of *QQS* were observed in 14 and 5 epiRILs, respectively (Figure 2D). This is consistent with Mendelian segregation of the highly methylated/lowly expressed Col-0 wt and lowly methylated/highly expressed Col-0 *ddm1* parental *QQS* epialleles (75%/25% expected because of backcrossing rather than selfing of the F1; *Chi^2^* = 0,017, p-value>0.05). Indeed, examination of the epi-haplotype obtained for 17 of these epiRILs [27] confirmed the wt or *ddm*-origin of the *QQS* locus in each case (data not shown). These results demonstrate therefore that, like many other *ddm1*-induced epialleles [28], [29], *QQS* hypomethylated epialleles can be stably inherited for at least eight generations and are not targets of paramutation.

### *QQS* Is under Autonomous Epigenetic Control

**Figure 3:**
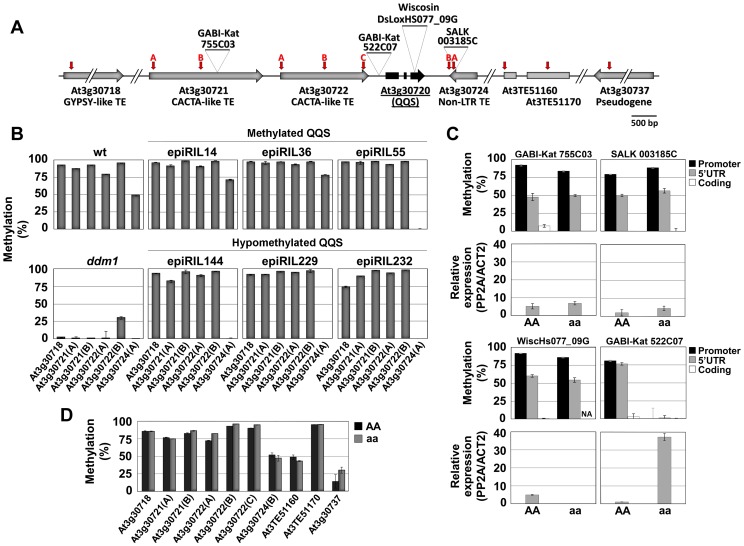
Epigenetic variation at *QQS* is determined by proximal sequences. (A) Schematic representation of the T-DNA/Transposon insertion sites (triangles; GABI-Kat 755C03, GABI-Kat 522C07, WiscDsLoxHs077_09G (WiscHs077_09G) and SALK 003185C) and McrBC-qPCR primer pairs used (vertical arrows; A, B and C represent different primer pairs designed for the same element). (B) DNA methylation levels of TEs flanking *QQS* in epiRILs that had inherited a wt or a *ddm1*-derived *QQS* epiallele. (C) DNA methylation and expression levels of *QQS* in lines carrying the T-DNA/transposon insertions represented in (A). (D) DNA methylation levels of TEs flanking *QQS* in the GABI-Kat 522C07 T-DNA insertion line. AA and aa represent wt and T-DNA homozygous individuals, respectively, coming from the selfing of one hemizygous (Aa) plant. NA, not analyzed. Error bars represent standard deviation observed in two technical replicates (B and D) or three biological replicates (C).

We next investigated the degree to which DNA methylation of *QQS* and of flanking TEs are independent from each other. To this end, we first analyzed DNA methylation patterns of TE sequences flanking *QQS* in a series of epiRIL with contrasted *QQS* epialleles. Unlike for *ddm1*-derived *QQS*, hypomethylation was not inherited for the three TEs located immediately upstream of the gene, as they did systematically regain wt DNA methylation levels (Figure 3A and 3B), presumably because of their efficient targeting by RNA-directed DNA methylation (RdDM) [28]. In addition, although the TE just downstream of *QQS* was always hypomethylated when inherited from *ddm1*, hypomethylation was also observed in one epiRIL that inherited the *QQS* region from the wt parent. Thus, there is no strict correlation between DNA methylation at *QQS* and this downstream TE. We next examined the effect of several T-DNA and transposon insertions located ∼3.1 kb or 153 bp upstream of the transcription start site (TSS), 653 bp downstream of the 3′UTR and within the second coding exon of *QQS*. Whereas three of these insertions had no effect on DNA methylation and expression levels of *QQS*, the T-DNA insertion located closest to the TSS was associated with a drastic reduction of DNA methylation of both the promoter and 5′UTR of the gene, as well as with an increase in *QQS* expression (Figure 3A and 3C). However, this insertion had no impact on DNA methylation of upstream and downstream TEs (Figure 3A and 3D). Taken together, these results suggest that epigenetic variation at *QQS* is most likely determined by sequences within the promoter and 5′UTR of the gene, not by the TEs that are located immediately upstream or downstream.

### *QQS* Exhibits Epigenetic Variation among Natural Accessions

**Figure 4:**
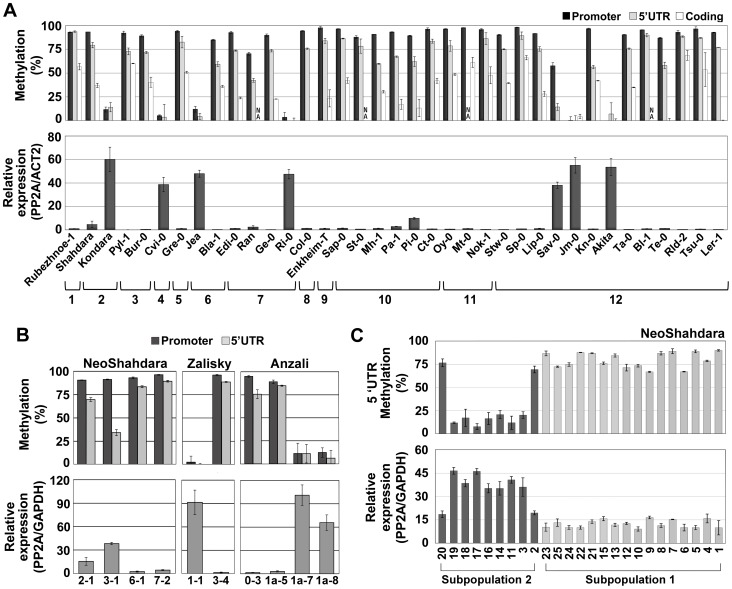
Epigenetic variation at *QQS* is frequent in nature. (A) DNA methylation and expression profile in natural accessions representing the worldwide diversity. Accessions are organized into clades 1 to 12 according to genetic relatedness [36]. NA, not analyzed. (B) DNA methylation and expression levels of *QQS* in plants grown from seeds directly collected in the Central Asian wild populations NeoShahdara, Zalisky and Anzali. For each line, one to three sibling plants were tested and gave similar results so that only one is represented per individual parent. (C) *QQS* epiallelic frequency among 25 NeoShahdara individuals. Plants analyzed here were obtained from seeds produced after two single seed descent generations. Error bars represent standard deviation observed between two (A – DNA methylation) or three (A – expression) biological replicates or two technical replicates (B and C).

We next investigated the possibility that *QQS* is subject to epigenetic variation in natural populations. To this end, we first analyzed *QQS* expression and DNA methylation in 36 accessions representing the worldwide diversity [30]. *QQS* was methylated and lowly expressed in 29 accessions, but unmethylated and highly expressed in seven accessions distributed over the entire geographic range (Figure 4A). This indicates that epigenetic variation at *QQS* is widespread in nature. In contrast, upstream and downstream TEs were consistently methylated in all accessions (Figure S4A and S4B), thus confirming that the epigenetic state at *QQS* is not determined by that of flanking TEs. We then sequenced a 2.8 kb interval encompassing the *QQS* gene and its flanking regions from the seven accessions carrying the hypomethylated/highly expressed epiallele as well as from three accessions carrying a methylated/lowly expressed epiallele. Although several SNPs and indels were identified (Figure S4C), no correlation between any specific sequence alterations and *QQS* DNA methylation or expression states could be established (Figure 4A). In addition, while Kondara and Shahdara have identical *QQS* sequences, they have contrasted DNA methylation/expression patterns at the locus (Figure 4A and Figure S4C), which provides further evidence that natural epiallelic variation at *QQS* is independent of local *cis*-DNA sequence polymorphisms and is thus most likely truly epigenetic. Analysis of a Cvi-0 vs. Col-0 Recombinant Inbred Line (RIL) population revealed in addition that *QQS* expression is controlled by a large-effect local-expression quantitative trait locus (local-eQTL; http://qtlstore.versailles.inra.fr/) [31]. This suggests that like the Col-0 wt and Col-0 *ddm1 QQS* epialleles (Figure 2D), the Cvi-0 hypomethylated *QQS* epiallele is stably inherited across multiple generations. This further demonstrates that epigenetic variation at *QQS* is not appreciably affected by sequence or DNA methylation polymorphisms located elsewhere in the genome and indicates also that *QQS* is not subjected to paramutation [29].

To validate experimentally the causal relationship between DNA methylation and repression at *QQS*, seedlings of several accessions were grown in the presence of the DNA methylation inhibitor 5-aza-2′-deoxycytidine (5-aza-dC). In the two accessions Col-0 and Shahdara, which harbor distinct methylated and lowly expressed *QQS* alleles, treatment resulted in reduced DNA methylation and increased expression of *QQS* (Figure S4D). In contrast, seedlings of Jea, Kondara and Cvi-0 accessions, all of which harbor a demethylated/highly expressed *QQS* allele, did not show further reduction of DNA methylation or increased expression when grown in the presence of the demethylating agent (Figure S4D). Moreover, whereas expression of *QQS* in F1 hybrids derived from crosses between Col-0 (methylated *QQS*) and Kondara (hypomethylated *QQS*), was always higher for the epiallele inherited from the hypomethylated parent, further confirming that *QQS* is not subjected to paramutation [29], treatment with 5-aza-dC reduced dramatically this expression imbalance, most likely as a consequence of demethylation of the Col-0-derived *QQS* allele (Figure S4E). Taken together, these results clearly demonstrate that DNA methylation at *QQS* is causal in repressing expression of the gene.

### Wild Populations from Central Asia Exhibit Epigenetic Variation at *QQS*

Finally, we asked whether epigenetic variation at *QQS* could be observed in natural settings or if such variation only emerged in the laboratory, where accessions are grown under controlled growth conditions. To this end, we analyzed *QQS* expression and DNA methylation in plants grown from seeds directly collected from wild populations in Tajikistan, Kyrgyzstan and Iran (NeoShahdara, Zalisky and Anzali populations, respectively). Widespread *QQS* epiallelic variation was observed, both between and within these diverse wild populations (Figure 4B). In addition, *QQS* epigenetic variation was examined in the offspring (after two single seed descent generations) of 25 NeoShahdara individuals. These individuals were randomly sampled among a single patch of several thousands of plants that presumably represent the direct descendants of the Shahdara accession. Based on 10 microsatellite markers and one InDel marker, two genetically distinct subpopulations could be identified. While *QQS* was highly methylated/lowly expressed in all 16 individuals of subpopulation #1, clear differences in DNA methylation and expression were detected among the 9 individuals of subpopulation #2 (Figure 4C). Whether epiallelic variation at *QQS* in subpopulation #2 reflects inherent fluctuations or an intermediary stage in the route to fixation of one of the two epiallelic forms remains to be determined.

## Discussion

*QQS* is a protein-coding gene that likely originated *de novo* in *A. thaliana* within a TE-rich region (Figure 1A). We have shown that this gene, which contains short repeat elements matching siRNAs (Figure 1B, Figure S1A and S1B), varies considerably in its DNA methylation and expression in the wild (Figure 4). We also show that these variations are heritable and independent of the DNA methylation status of neighboring TEs or of DNA sequence variation, either in *cis* or *trans* (Figure 2 and Figure 3, Figures S2 and S4). Thus, we can conclude that *QQS* is a hotspot of epigenetic variation in nature. Consistent with this, *QQS* is among the few genes for which spontaneous DNA methylation variation was observed in Col-0 mutation accumulation lines [13].

Cytosine methylation at *QQS* concerns CG, CHG and CHH sites, which is the pattern expected for sequences with matching siRNAs (Figure 1B, Figure S1B). All three types of methylation sites likely contribute to silencing of *QQS*, as judged by the reactivation of *QQS* in the *met1*, *ddm1*, *ddc* and *rdr2* mutant backgrounds (Figure S1C; [24], [25]). Yet, among the different DNA methyltransferases targeting DNA methylation at *QQS*, *MET1* may play a more prominent role, given that DNA methylation at this locus is only fully erased in *met1* mutant plants [25]. *QQS* demethylated epiallelic variants may thus preferentially arise through spontaneous [13] or stress-induced [10] defects in DNA methylation maintenance and be stably inherited for multiple generations as a result of the concomitant loss of matching siRNAs, which would prevent efficient remethylation and silencing of the gene [28], [29]. Indeed, although we could not detect *QQS* siRNAs by Northern blot analysis, presumably because of their low abundance, deep sequencing data indicate that they accumulate less in *met1* mutant plants than in wild type Col-0 [25].

Few genes have been shown so far to be subject to heritable epigenetic variation in *A. thaliana* [5]–[8], [13], [14], [32] and *QQS* is unique among these in exhibiting this type of variation in nature (Figure 4). This therefore raises the question as to what distinguishes *QQS* from other genes, such as *FWA*, for which epigenetic variation can be readily induced in the laboratory in advanced generations of *ddm1* and *met1* mutant plants [5], [33], but for which this type of variation is not observed among accessions [11], [34]. One possibility is that unlike *QQS* epivariants, *fwa*-hypomethylated epialleles are strongly counter-selected because of their potentially maladapted phenotype, namely late flowering [5]. Consistent with this explanation, epiallelic variation with no phenotypic consequences has been documented at *FWA* in other Arabidopsis species. In these cases, however, inheritance across multiple generations has not been rigorously tested [35]. Another possibility is that *de novo* originated genes, such as *QQS*, are particularly prone to heritable epigenetic variation. This is a reasonable assumption considering that these genes tend to lack proper regulatory sequences initially, unlike new gene duplicates, which by definition come fully equipped [21]. In turn, given that epigenetic variation enables genes to adjust their expression in a heritable manner much more rapidly than through mutation while preserving the possibility for rapid reversion, it could prove particularly beneficial in the case of genes that are created from scratch. Once the most adaptive expression state is reached, it could then become irreversibly stabilized (i.e. genetically assimilated) through DNA sequence changes. Although speculative, this proposed scenario could be highly significant given the recent discovery that *de novo* gene birth may be more prevalent than gene duplication [23].

## Materials and Methods

### Plant material and growth conditions

*A. thaliana* accessions were obtained from the INRA Versailles collection (dbsgap.versailles.inra.fr/vnat/, www.inra.fr/vast/collections.htm) [30], [36], [37]. Insertion lines were obtained from the GABI-Kat at University of Bielefeld, Germany (GABI-Kat 755C03 and 522C07) [38], the ABRC at Ohio State University (SALK 003195C) and University of Wisconsin, Madison, US (WiscDsLoxHs077_09) [39]. Seeds of *ddm1-2* [40], *rdr2-1* [41] and *ddm1*-derived epiRIL lines [26] were provided by V.Colot. NeoShahdara individuals were genotyped with 10 microsatellite markers (NGA8, MSAT2.26, MSAT2.4, NGA172, MSAT3.19, ICE3, MSAT3.1, MSAT3.21, MSAT4.18, ICE5; http://www.inra.fr/vast/msat.php) and one InDel marker in MUM2 gene (MUM2_Del-LP TGGTCGTTATTGGGTCTCGT, MUM2 Del-RP TTAAGAACGCCCGAGGAATA). For expression and DNA methylation assays, seedlings were grown in vitro (MS/2 media supplemented with 0,7% sucrose) for eight days in a culture room (22°C, 16 hours light/8 hours dark cycle, 150 µmol s−1 m−2). Treatment with 5-aza-2′-deoxycytidine was performed as described in [8].

### RT–qPCR analysis of *QQS* expression

Total RNA was isolated as described in [42] and cDNA was synthetized using oligo(dT) primers and IMPROM II reverse transcriptase (Promega). Real time PCR reactions were run on an Applied Biosystems 7500 Real-Time PCR System using Platinum SYBR green (Invitrogen). *QQS* expression levels relative to *Actin2*/*PP2A* or *PP2A*/*GAPDH* internal references were calculated using the formula (2^- (Ct *QQS* – mean Ct internal references)^)*100. Primers are listed in Table S1.

### Analysis of DNA methylation by McrBC–qPCR

Total DNA was isolated using Qiagen Plant DNeasy kit following the manufacturer's recommendations. Digestion was carried out overnight at 37°C with 200 ng of genomic DNA and 2 to 8 units of McrBC enzyme (New England Biolabs). Quantitative PCR was performed as described above on equal amounts (2 ng) of digested and undigested DNA samples using the primers described in Table S1. Results were expressed as percentage of molecules lost through McrBC digestion (1-(2^-(Ct digested sample - Ct undigested sample)^))*100. As a control, the percentage of DNA methylation for *At5g13440*, which is unmethylated in wt, was estimated in all analyses.

### Allele-specific expression assays

To assess the relative contribution of each allele to the population of mRNA in F1 individuals from reciprocal crosses between Col and Kondara, a single pyrosequencing reaction using the primers QQS_pyro_F1 (PCR) - TCAAAATGAGGGTCATATC ATGG, QQS_pyro_R1-biotin (PCR) - ATTGGATACAATGGCCCTATAACT and QQS_pyro_S1 (Pyrosequencing) - GATATTGGGCCTTATCAC was set up on a SNP polymorphic between the *QQS* parental coding sequences (Figure S4C; position +285). Pyrosequencing was performed on F1 cDNA, as well as on 1/1 pools of parents cDNA to establish the allelic contribution to *QQS* expression. F1 genomic DNA is used as pyrosequencing control to normalize against possible pyrosequencing biases. Anything significantly driving allele-specific expression in hybrids is by definition acting in *cis*, since F1 nuclei contain a mix of all *trans*-acting factors [43], [44].

### Comparative genome hybridization (CGH)

CGH experiments were performed for Col-0* vs. Col-0 using Arabidopsis whole-genome NimbleGen tiling arrays [45]. The normalmixEM function of the mixtools package on R was used to found the normal distribution for the distribution of the Col-0*/Col-0 ratio with an expected number of gaussians of two. A Hidden Markov model [46] was used to find regions with copy number variation.

### Analysis of genome wide DNA methylation (MeDIP–Chip)

DNA was extracted using DNeasy Qiagen kit and MeDIP-chip was performed on 1.8 µg of DNA as previously described in [47]. The methylated tiles were identified using the ChIPmix method [48]. Probes methylated in one line only (Col-0 or Col-0*) were used to create domains. Domains contain at least three consecutive or nearly consecutive (400 nt min, with one gap of 200 nt max) tiles with identical methylation patterns.

### Overall codon-based Z-test of purifying selection

Available *QQS* coding-sequences (464 different accessions) were downloaded from the “Salk Arabidopsis 1001 Genomes” database (http://signal.salk.edu/atg1001/index.php). *A. suecica QQS* sequence (coming from the *A. thaliana* genome of this allotetraploid [49]) was also included in the analysis. The aligned sequences were used to calculate the probability of rejecting the null hypothesis (H_0_) of strict-neutrality (dN = dS; where dN = number of nonsynonymous and dS = number of synonymous substitutions per site) in favor of the alternative hypothesis of purifying selection (H_A_; dS>dN). The analysis was done using the MEGA5 software under the Nei-Gojobori method [50] with the variance of the difference calculated by the bootstrap method with 100 replicates. Our overall analysis of 465 sequences rejected H_0_ in favor of H_A_ (dN/dS = 0.5868; p-value<0.045).

## Supporting Information

Figure S1 *QQS* expression is negatively correlated with DNA methylation. (A) Schematic representation of the tandem repeats present at the *QQS* promoter and 5′UTR region. (B) Distribution of DNA methylation at the *QQS* promoter and 5′UTR sequences. Data is presented as the total number of unmethylated (C) and methylated cytosines (5 mC) in the three sequence contexts (CG, CHG and CHH, H = A, T or C) for both DNA strands. DNA methylation data are from [25]. (C) Assessment of *QQS* DNA methylation level and transcript accumulation in seedlings of *ddm1-2* and *rdr2-1* mutants. Error bars represent standard deviation between two (DNA methylation) or three (expression) biological replicates.
(TIF)

Figure S2 Genome-wide analyses of Col-0 and Col-0*. (A) Comparative genomic hybridization (CGH) analysis of Col-0* vs. Col-0 represented as the average of the log 2 ratio of the signal for the INPUT Col-0* over INPUT Col-0. A single normal distribution is observed using the normalmixEM function of the mixtools package on R with an expected number of Gaussians of two. The CGH analyses of Col-0* and Col-0 show no decrease or increase in copy number in Col-0*, suggesting that they correspond to the same accession. In contrast, CGH of Col-0 vs. Cvi and Col-0 vs. C24 revealed 6.0 and 5.5% of tiles with significant copy number variation, respectively [Moghaddam, et al (2011)]. (B–D) Methylated DNA Imunoprecipitation assays. Representation of the proportion of domains that are methylated (B) in only one replicate of Col-0 or in both, (C) in only one replicate of Col-0* or in both and (D) in only Col-0 or Col-0* or in both. A total of 86% of the domains are methylated in both Col-0 and Col-0*, which is similar to the result obtained for two biological replicates of Col-0 or of Col-0* (89% and 91% of the domains methylated in the two replicates, respectively). These results indicate that the methylomes of Col-0* and Col-0 are only marginally more dissimilar from each other as they are from their biological replicates. [Moghaddam A.B, Roudier F, Seifert M, Berard C, Magniette MLM, et al. (2011) Additive inheritance of histone modifications in *Arabidopsis thaliana* intra-specific hybrids. Plant J 67: 691–700. doi: 10.1111/j.1365-313X.2011.04628.x].
(TIF)

Figure S3 Schematic representation of the experimental design used to analyze *QQS* expression and DNA methylation state in single seed descent lines (named Line A, Line B, Line C and so on) at the S1 and S2 generations.
(TIF)

Figure S4 DNA methylation levels of *QQS* correlate negatively with *QQS* expression in natural accessions. (A) Schematic representation of a 30 kb genomic region encompassing *QQS*. Red arrows indicate McrBC-qPCR primer pairs used to determine DNA methylation levels of TEs flanking *QQS*; A, B and C represent different primer pairs designed for the same element. (B) DNA methylation levels of TEs flanking *QQS* in Col-0 (methylated *QQS* epiallele), Jea, Ri-0, Sav-0, Cvi-0, Kondara, Jm-0 and Akita (hypomethylated *QQS* epiallele) accessions. ‘NA’: not analyzed; ‘ND’: not determined (presumably because of DNA sequence polymorphisms preventing primer annealing). Error bars represent standard deviation observed in two technical replicates. (C) DNA sequence polymorphisms at the *QQS* locus and flanking region in accessions carrying methylated and hypomethylated *QQS* epialleles. The region analyzed comprises 1.5 kb upstream and 0.6 kb downstream of the *QQS* transcription initiation and termination sites, respectively. Nucleotide positions are numbered relative to the *QQS* translation initiation site (Position +1). Methylated accessions (Col-0, Pyl-1, Mh-1 and Shahdara) are shown in red and hypomethylated accessions (Kondara, Cvi-0, Jea, Ri-0, Sav-0, Jm-0 and Akita) in black. (D) Effect of the methylation inhibitor 5-aza-dC on DNA methylation and expression of *QQS*. Error bars at represent standard deviation observed in at least 3 biological replicates. (E) Pyrosequencing quantification of allele-specific expression of *QQS* in F1 seedlings derived from a cross between Col-0 and Kondara and grown with or without 5-aza-dC. Data is expressed as the % of total transcripts originating from the Kondara allele (top panel). DNA methylation level in the same two pools of F1 seedlings (bottom panel). Error bars represent standard deviation observed in two technical replicates.
(TIF)

Table S1 Primer list.
(DOCX)
