# Supplementary figures and images for: Extensive Natural Epigenetic Variation at a De Novo Originated Gene
Source: PLoS Genet. 2013 Apr 11;9(4):e1003437. doi: 10.1371/journal.pgen.1003437 (PMC3623765; doi:10.1371/journal.pgen.1003437)

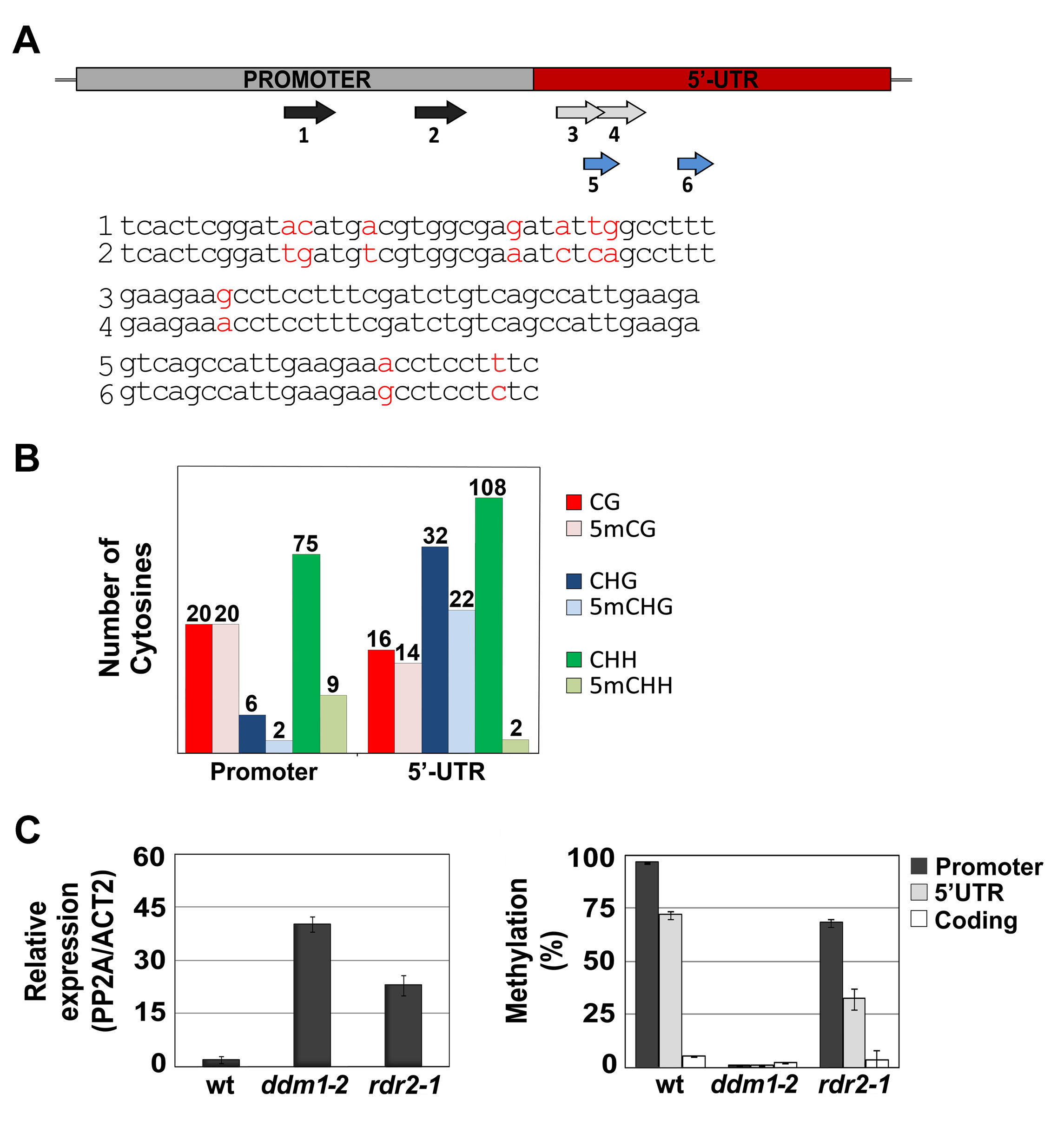

Supplement: Figure S1 — QQS expression is negatively correlated with DNA methylation. (A) Schematic representation of the tandem repeats present at the QQS promoter and 5′UTR region. (B) Distribution of DNA methylation at the QQS promoter and 5′UTR sequences. Data is presented as the total number of unmethylated (C) and methylated cytosines (5 mC) in the three sequence contexts (CG, CHG and CHH, H = A, T or C) for both DNA strands. DNA methylation data are from [25]. (C) Assessment of QQS DNA methylation level and transcript accumulation in seedlings of ddm1-2 and rdr2-1 mutants. Error bars represent standard deviation between two (DNA methylation) or three (expression) biological replicates. (TIF) [file pgen.1003437.s001.tif]

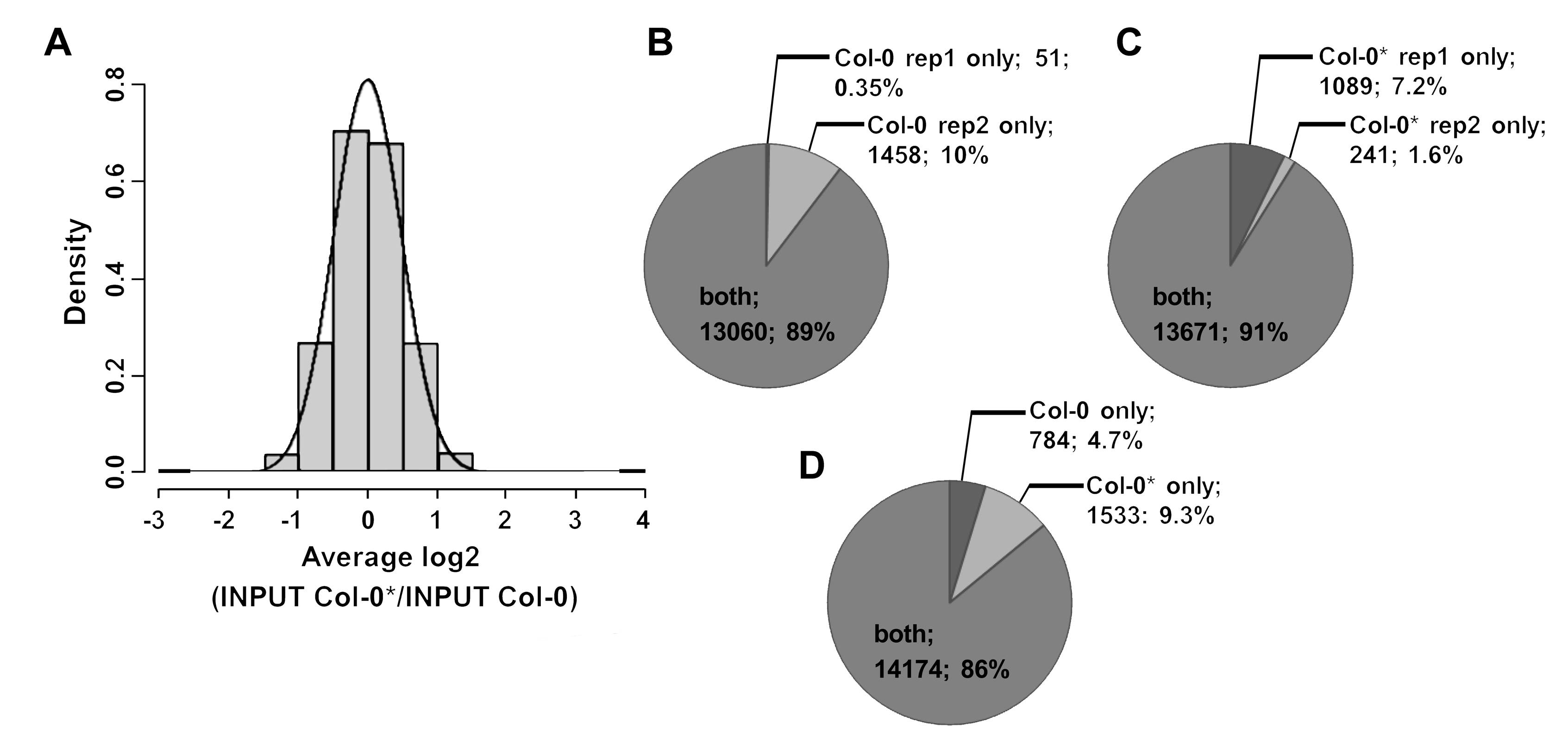

Supplement: Figure S2 — Genome-wide analyses of Col-0 and Col-0*. (A) Comparative genomic hybridization (CGH) analysis of Col-0* vs. Col-0 represented as the average of the log 2 ratio of the signal for the INPUT Col-0* over INPUT Col-0. A single normal distribution is observed using the normalmixEM function of the mixtools package on R with an expected number of Gaussians of two. The CGH analyses of Col-0* and Col-0 show no decrease or increase in copy number in Col-0*, suggesting that they correspond to the same accession. In contrast, CGH of Col-0 vs. Cvi and Col-0 vs. C24 revealed 6.0 and 5.5% of tiles with significant copy number variation, respectively [Moghaddam, et al (2011)]. (B–D) Methylated DNA Imunoprecipitation assays. Representation of the proportion of domains that are methylated (B) in only one replicate of Col-0 or in both, (C) in only one replicate of Col-0* or in both and (D) in only Col-0 or Col-0* or in both. A total of 86% of the domains are methylated in both Col-0 and Col-0*, which is similar to the result obtained for two biological replicates of Col-0 or of Col-0* (89% and 91% of the domains methylated in the two replicates, respectively). These results indicate that the methylomes of Col-0* and Col-0 are only marginally more dissimilar from each other as they are from their biological replicates. [Moghaddam A.B, Roudier F, Seifert M, Berard C, Magniette MLM, et al. (2011) Additive inheritance of histone modifications in Arabidopsis thaliana intra-specific hybrids. Plant J 67: 691–700. doi: 10.1111/j.1365-313X.2011.04628.x]. (TIF) [file pgen.1003437.s002.tif]

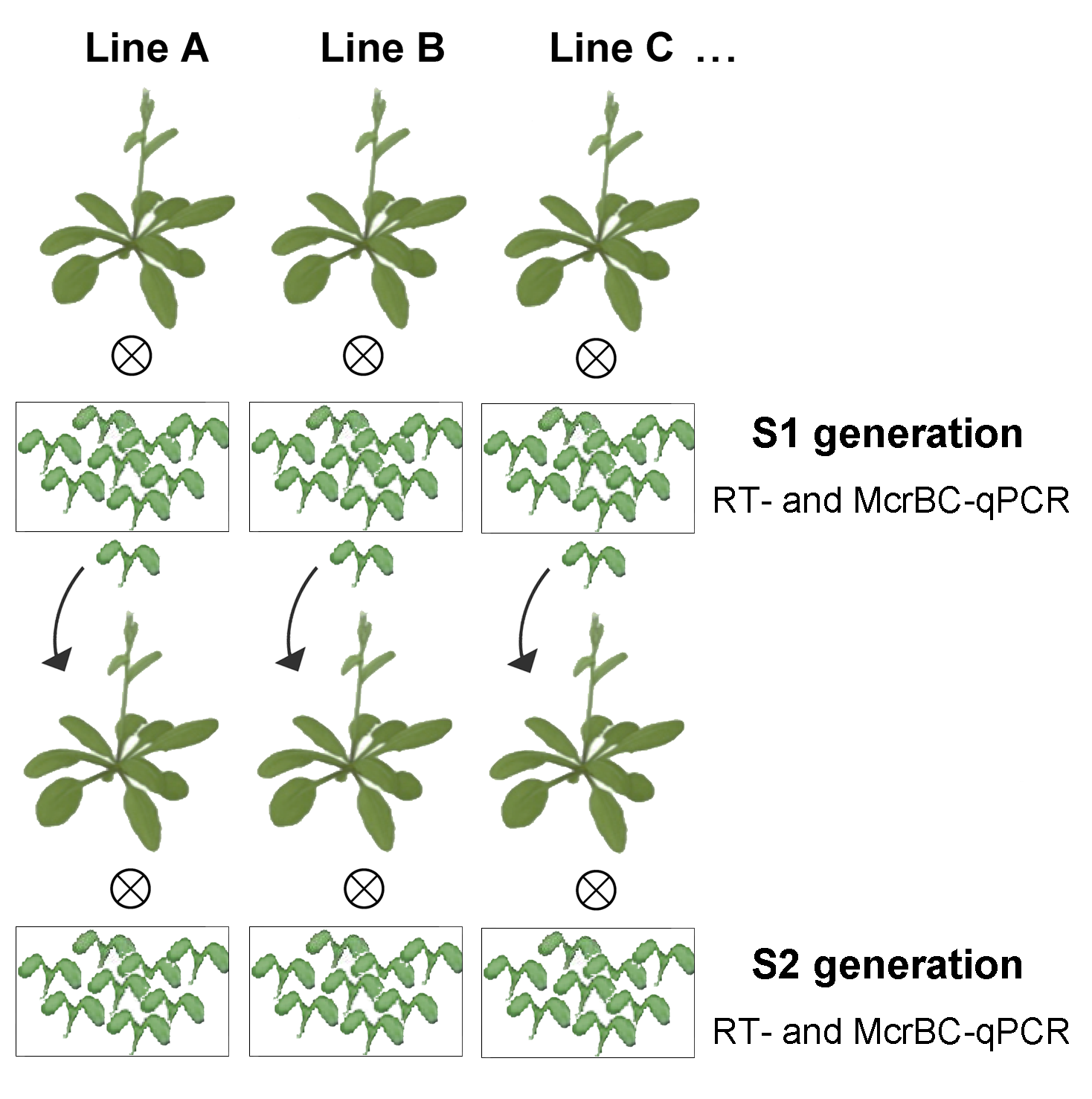

Supplement: Figure S3 — Schematic representation of the experimental design used to analyze QQS expression and DNA methylation state in single seed descent lines (named Line A, Line B, Line C and so on) at the S1 and S2 generations. (TIF) [file pgen.1003437.s003.tif]

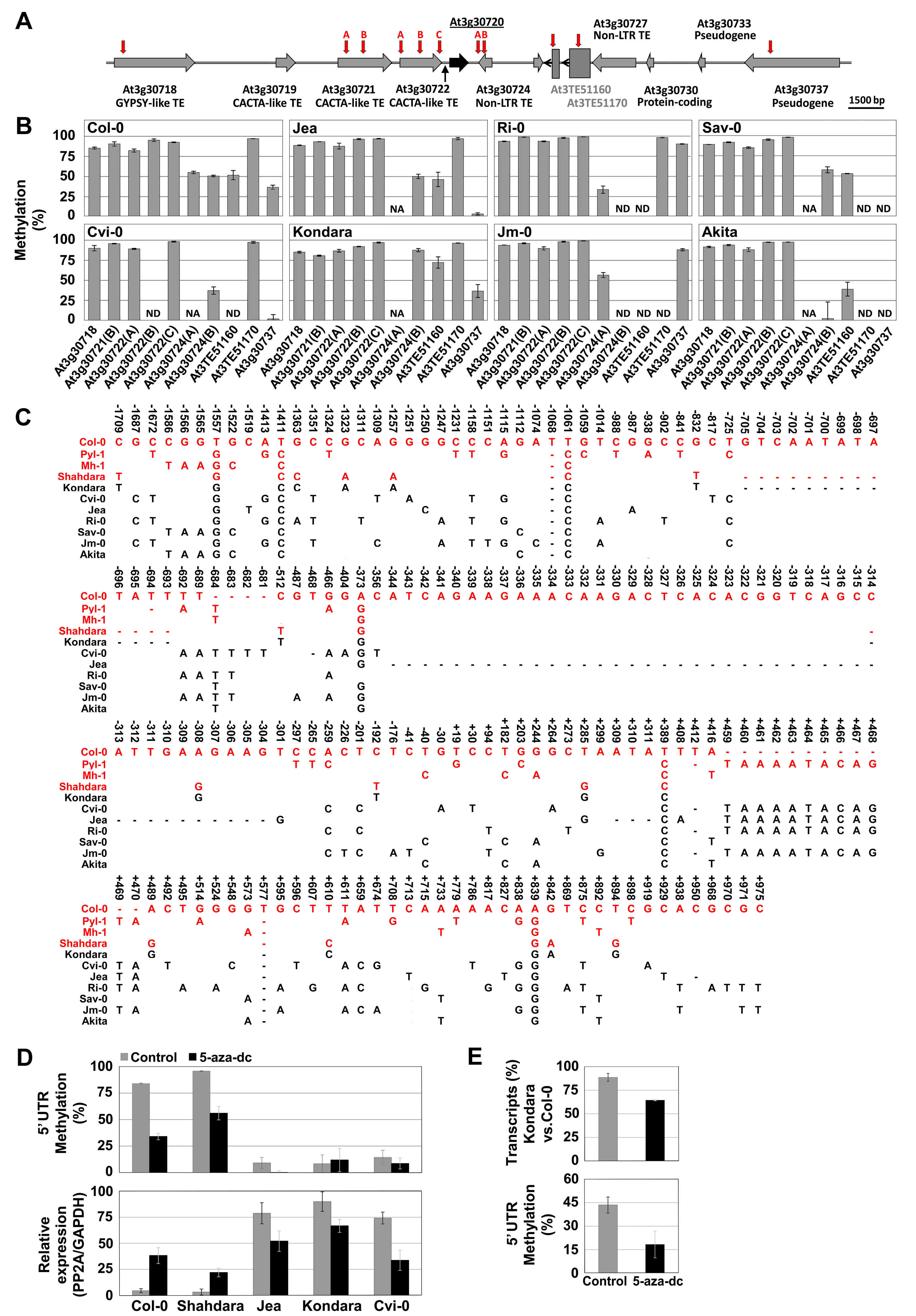

Supplement: Figure S4 — DNA methylation levels of QQS correlate negatively with QQS expression in natural accessions. (A) Schematic representation of a 30 kb genomic region encompassing QQS. Red arrows indicate McrBC-qPCR primer pairs used to determine DNA methylation levels of TEs flanking QQS; A, B and C represent different primer pairs designed for the same element. (B) DNA methylation levels of TEs flanking QQS in Col-0 (methylated QQS epiallele), Jea, Ri-0, Sav-0, Cvi-0, Kondara, Jm-0 and Akita (hypomethylated QQS epiallele) accessions. ‘NA’: not analyzed; ‘ND’: not determined (presumably because of DNA sequence polymorphisms preventing primer annealing). Error bars represent standard deviation observed in two technical replicates. (C) DNA sequence polymorphisms at the QQS locus and flanking region in accessions carrying methylated and hypomethylated QQS epialleles. The region analyzed comprises 1.5 kb upstream and 0.6 kb downstream of the QQS transcription initiation and termination sites, respectively. Nucleotide positions are numbered relative to the QQS translation initiation site (Position +1). Methylated accessions (Col-0, Pyl-1, Mh-1 and Shahdara) are shown in red and hypomethylated accessions (Kondara, Cvi-0, Jea, Ri-0, Sav-0, Jm-0 and Akita) in black. (D) Effect of the methylation inhibitor 5-aza-dC on DNA methylation and expression of QQS. Error bars at represent standard deviation observed in at least 3 biological replicates. (E) Pyrosequencing quantification of allele-specific expression of QQS in F1 seedlings derived from a cross between Col-0 and Kondara and grown with or without 5-aza-dC. Data is expressed as the % of total transcripts originating from the Kondara allele (top panel). DNA methylation level in the same two pools of F1 seedlings (bottom panel). Error bars represent standard deviation observed in two technical replicates. (TIF) [file pgen.1003437.s004.tif]
