# Supplementary material for: Extensive Natural Epigenetic Variation at a De Novo Originated Gene
Source: PLoS Genet. 2013 Apr 11;9(4):e1003437. doi: 10.1371/journal.pgen.1003437 (PMC3623765; doi:10.1371/journal.pgen.1003437)
Supplement: Table S1 — Primer list. (DOCX) [file pgen.1003437.s005.docx]

| Table S1. Primer List | | | | |
| --- | --- | --- | --- | --- |
| **Expression analysis** | |  |  |  |
| **AGI** | **Primer pair** | **Forward primer** | **Reverse primer** | **Experiments** |
| *At3g30720 (QQS)* | QQS_1 | AAGACCAATAGAGAGCAGGAA | CCTGATGTAGAAGTGTGAGG | All, with the exception of WiscDsLoxHs077_09G genotype on Figure 3 |
|  | QQS_2 | CACTTCTACATCAGGTGTCG | AAGGCCCAATATCAGTAGTTG | WiscDsLoxHs077_09G on Figure 3 |
| *AT1G13320 (PP2A)* | PP2A_1 | CATGTTCCAAACTCTTACCTG | GTTCTCCACAACCGCTTGGT | Figures 2, 3, 4A, S1 |
|  | PP2A_2 | TTTGTGAAGCTGTAGGACCG | CGAGTTCAGGGTTTAAAATGCG | Figures 4B, 4C and S4 |
| *AT3G18780 (Actin2)* | ACT2 | GTACAACCGGTATTGTGCTGG | CAAGGTCAAGACGGAGGATG | Figures 2, 3, 4A, S1 |
| *At1g13440(GAPDH)* | GAPDH | TTGGTGACAACAGGTCAAGC | AAACTTGTCGCTCAATGCAATC | Figures 4B, 4C and S4 |
| **DNA methylation quantification** | | | | |
| **AGI** | **Primer pair** | **Forward primer** | **Reverse primer** | **Experiments** |
| *At3g30720(QQS)* | Promoter (F1A/R1A) | tcactcggattgatgtcgtg | AGGAGACGAAACAGACAAATC | Figures 2, 3 (with the exception of GABI-Kat_522C07), 4 (Col-0, Gre-0, Ct-1; Enkheim-0, Jea, Ler-1, Nok-1, Pa-1, Pi-0, Sp-0, Bur-0, Edi-0, Te-0, Mh-1, Sav-0, Mt-0, Akita, Kondara, Shahdara, Tsu-0, NeoShahdara, Zalisky and Anzali) and S1 |
|  | Promoter (F1A/R1B) | tcactcggattgatgtcgtg | AGGAGATGAAACAGACAAATC | Figure 4 (Cvi-0, Bl-1, Bla-1, Ge-0, Pyl-1, Oy-0, Jm-0, Kn-0, Lip-0, Rubezhoe, Sap-0, Ta-0, Rld-2, Stw-0) |
|  | Promoter (F1B/R1B) | GCCAATTAGAATGTTTCACTCG | AGGAGATGAAACAGACAAATC | Figure 4 (Ran, St-0) |
|  | Promoter (F1C/R1A) | TCCAAGCTTGCCAAAACGATC | AGGAGACGAAACAGACAAATC | Figure 3 (GABI-Kat_522C07) |
|  | 5’-UTR (F2A/R2A) | tctgtcagccattgaagaaac | GATAAGGTTTGGGTACAGATC | Figures 2, 3, 4 (Col-0, Ri-0, Gre-0, Ct-1, Enkheim-T, Ler-1, Nok-1, Pa-1, Pi-0, Ran, Sp-0, Bur-0, St-0, Mh-1, Sav-0, Ta-0, Mt-0, Akita, Kondara, Shahdara, Tsu-0, NeoShahdara, Zalisky and Anzali), S1 and S4 |
|  | 5’-UTR (F2A/R2B) | tctgtcagccattgaagaaac | GATAAGGGTTGGGTACAGATC | Figure 4 (Cvi-0, Bl-1, Bla-1, Ge-0, Pyl-1, Edi-0, Oy-0, Te-0, Jm-0, Lip-0, Rubezhnoe, Sap-0, Rld-2, Stw-0) |
|  | 5’-UTR (F2B/R2B) | TCTGTCAGCCATTGAAGAAGC | GATAAGGGTTGGGTACAGATC | Figure 4 (Kn-0) |
|  | 5’-UTR (F2B/R2C) | TCTGTCAGCCATTGAAGAAGC | GATAAGGTTTGGGCACAGATC | Figure 4 (Jea) |
|  | Coding (F3A/R3A) | AAGGTTCATTTTGCCTCACAC | AAGGCCCAATATCAGTAGTTG | Figures 2, 3 (with the exception of WiscDsLoxHs077_09G), 4 (Col-0, Gre-0, Ri-0, Bla-1, Ct-1, Enkheim-T, Ge-0, Jea, Ler-1, Nok-1, Pyl-1, Sp-0, Bur-, Edi-0, Oy-0, Te-0, Jm-0, Kn-0, Lip-0, Rubezhnoe, Sap-0, Ta-0, Mt-0, Kondara, Rld-2, Shahdara, Stw-0, Tsu-0, NeoShahdara, Zalisky and Anzali) and S1 |
|  | Coding (F3B/R3A) | AAGGTTCATTCTGCCTCACAC | AAGGCCCAATATCAGTAGTTG | Figure 4 (Pa-1, Pi-0, Mh-1, Sav-0, Cvi-0, Akita) |
| *At5g13440* | At5g13440 | ACAAGCCAATTTTTGCTGAGC | ACAACAGTCCGAGTGTCATGGT | All |
| *At3g30722* | At3g30722(A) | GCCGTAGTAACCGTCAGGAA | AGACATTTTATTCTGTTAAGTGG |  |
|  | At3g30722(B) | CTGCTAGAATGGGGTTCATC | CCTCCATAGTGGCGAATCAC |  |
|  | At3g30722(C) | GTTAGACTACAAGTACCAACTC | AAGAGTTGCAGGATCCGTCG |  |
| *At3g30721* | At3g30721(A) | CTCTGGAGCATCAATTAGTTTG | ACTTCAAATCCATACCTCTGAT |  |
|  | At3g30721(B) | GAGAAACCTTCGTCTTGGTC | GGGATCAACATAGTCAACATG | As indicated on Figures 3 and S4 |
| *At3g30718* | At3g30718 | GTCTAGATATCCAGGGGATG | CTCTGAACTATCAACATGTGC |  |
| *At3g30724* | At3g30724(A) | tcctgtgctattgatactcac | GACAAAACAAGTCTGATCGATG |  |
|  | At3g30724(B) | tcctgtgctattgatactcac | GATGTTTTTGCAGTCAATGAAAC |  |
| *AT3TE51160* | AT3TE51160 | ctttacttacaaagtagatgagc | CTCGAGATTGACTCTTTTGAG |  |
| *At3TE51170* | At3TE51170 | cctgtgatataccgtctcgt | GGTCGATAGTAATACGAGAGA |  |
| *At3g30737* | At3g30737 | agtgtctcgaacgtgtgtcg | GTTACAGAAGATTCCATTTGTG |  |
